# Reliability of the Digital Image Analysis Program Mdesk for Assessing Acetabular Component Positioning on Plain Radiographs Following Total Hip Arthroplasty: A Multicenter Inter- and Intra-observer Reliability Study

**DOI:** 10.1007/s43465-025-01419-0

**Published:** 2025-06-03

**Authors:** Anne Mette Stausholm, Thomas Jakobsen, Anders El-Galaly, Rasmus Elsoe, Martin Magnèli, Henrik Malchau, Poul Torben Nielsen

**Affiliations:** 1 Department of Orthopedic Surgery, Aalborg University Hospital, 18-22 Hobrovej, 9000 Aalborg, Denmark; 2 Department of Clinical Sciences, Division of Orthopedics, Danderyd Hospital, Karolinska Institutet, Stockholm, Sweden; 3 Harris Orthopedic Laboratory, Massachusetts General Hospital, Boston, USA; 4 Department of Orthopedic Surgery, Harvard Medical School, Boston, USA

**Keywords:** Mdesk, Reliability, Postoperative analysis, THA, Radiology, Digital, Hip replacement, Hip, Validation, Clinical monitoring

## Abstract

**Background:**

In total hip arthroplasty (THA), correct fixation and positioning of both the acetabular and femoral components are prerequisites for successful outcomes. Continuous registration and analysis of these components from postoperative X-rays is important in quality monitoring after THA. Reliable measurements for positioning assessments are essential in this quality monitoring. The objective of this study was to evaluate the inter- and intra-observer reliability of Mdesk for measuring acetabular component positioning in THA.

**Methods:**

This was a multicenter inter- and intra-observer reliability study conducted across multiple countries, with 102 patients assessed from 2008 to 2012. Patients with osteoarthritis treated with unilateral total hip arthroplasty (THA) were enrolled in the study. Postoperative AP pelvic and cross-table lateral radiographs were analyzed using the digital image analysis program Mdesk version 4.0. The inter- and intra-tester reliability was assessed by calculating interclass correlation coefficients (ICC) with 95% confidence intervals (CI).

**Results:**

The intraobserver reproducibility of all measurements was high (ICC > 0.80). The interobserver reliability was high for all measurements (ICC > 0.74), except for Δ lateral offset and cup protrusion, which was only moderate (ICC = 0.665 and ICC = 0.614 and 0.600).

**Conclusion:**

Mdesk showed high reliability in measuring key acetabular parameters, suggesting it is a viable tool for THA postoperative monitoring.

## Introduction

Total hip arthroplasty (THA) is one of the most successful orthopedic surgical procedures [1]. THA offers significant pain relief, improved quality of life, and increased mobility [1]. However, complications following THA are not uncommon and are associated with worse patient satisfaction. The leading causes of complications and early failures are dislocation, aseptic loosening, wear, periprosthetic fractures, and infection. [2]

The etiology of early complications of primary THA is multifactorial. It is generally accepted that correct implantation and positioning of the acetabular and femoral components is a prerequisite for both optimal function and longevity of the implant [4–6]. The significance of incorrect cup sizing [4, 5], incomplete cup seating [6], cup protrusion [7], and incorrect cup positioning [8] on hip biomechanics are commonly reported. Altered biomechanics, such as impingement [9], dislocation, squeaking of ceramic-on-ceramic components [10], and osteolysis [11, 12] may influence outcome and patients overall satisfaction. Consequently, evaluating cup positioning from postoperative X-rays is essential to quality monitoring after THA.

Measuring implant positing following THA is performed using several methods. Hardcopy analog X-rays have previously been used. However, the transition to digital X-ray systems in most hospitals has made this method obsolete. At present, several digital pre-operative and post-operative image measurement systems are available. Mahmood et al. [13] evaluated the validity of the Sundsvall method for femoral offset measurements. Callanan et al. [14] used Martell Hip Analysis Suite to calculate the cup inclination and version angles. Grammatopoulos et al. [15] measured the inclination and version angles using the Ein-Bild-Roentgen-Analysis (EBRA) software. Bombaci et al. [16] determined the hip rotation center from landmarks in the digital pelvic radiograph. Another commonly used digital measurement system is the Mdesk, which was developed for pre-planning surgery and post-operative analysis.

Before the inclusion of digital measurement properties in clinical practice, their validity and reliability must be documented. Several studies have reported on the reliability of selected acetabular measurements; however, existing literature lacks information on the reliability of complete cup positioning measurements for the Mdesk system [14, 15, 17].

The objective of this study was to evaluate the inter- and intra-observer reliability of Mdesk for measuring acetabular component positioning in THA.

## Methods

### Study Design

This cross-sectional, reliability-based study was conducted across multiple centers. Study approval was obtained from the local Institutional Review Board or the Ethical Committee at all included centers. The study approval included that informed consent was not required and all ICMJE standards are meet.

The primary outcome of the present study was inter- and intra-tester reliability measured by an interclass correlation coefficients (ICC) with 95% confidence intervals (CI) and absolute agreement.

### Participants

Included were patients with hip osteoarthritis who had been treated with unilateral THA from 2008 to 2012. All patients were among participants in a multicenter study between 17 centers in 8 countries (USA, Mexico, Norway, Sweden, Denmark, the Netherlands, UK, and Spain) established in 2007 to monitor the safety and clinical outcomes of new biomaterials [18].

The original study population established in 2007 consisted of 977 patients. 267 patients, who had a contralateral THA or had a bilateral surgery performed, were excluded from the present study. Due to supplemental ethical approval, patients from three countries (Sweden, Denmark, and the USA) were eligible for the present study. A total of 102 patients who had been treated in Sweden (Sahlgrenska and Uddevalla), Denmark (Hvidovre), and the USA (New York) were included.

Hence, of the 977 patients included in the original study, a total of 102 patients were included in the present study.

### Outcomes

**Fig. 1:**
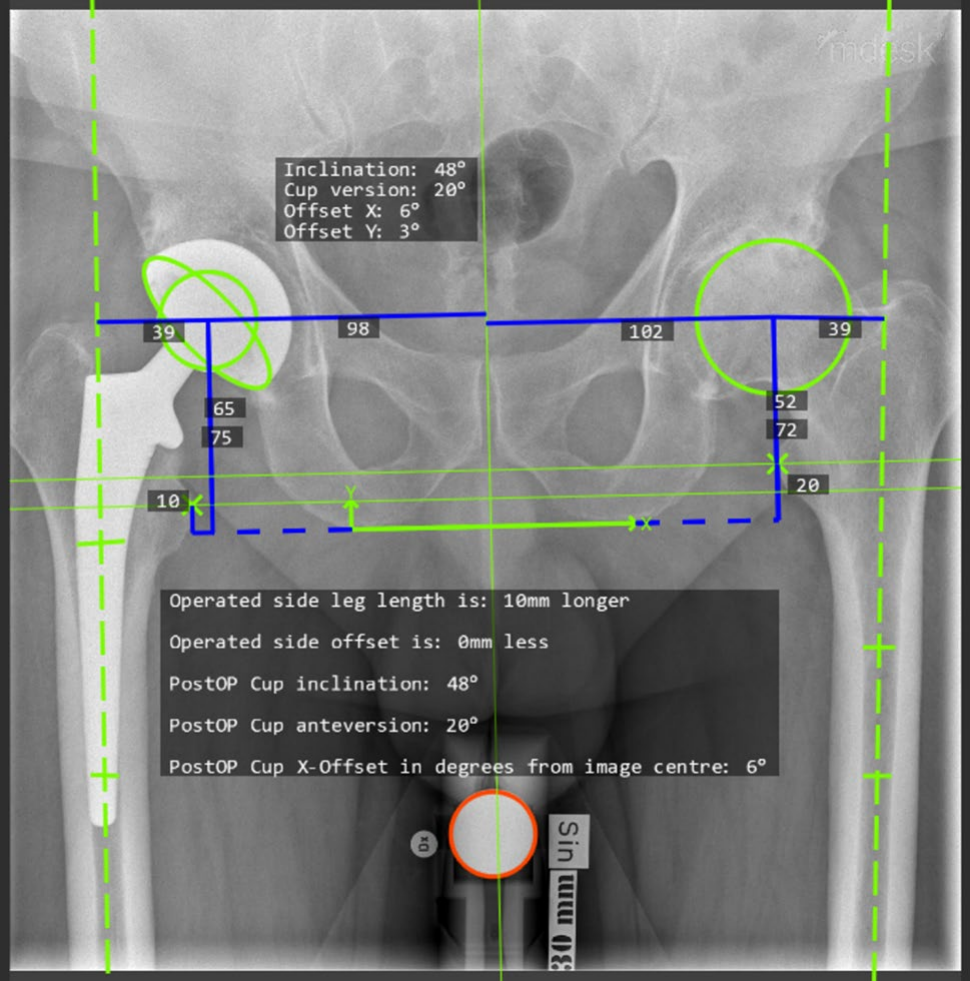
Mdesk

**Fig. 2:**
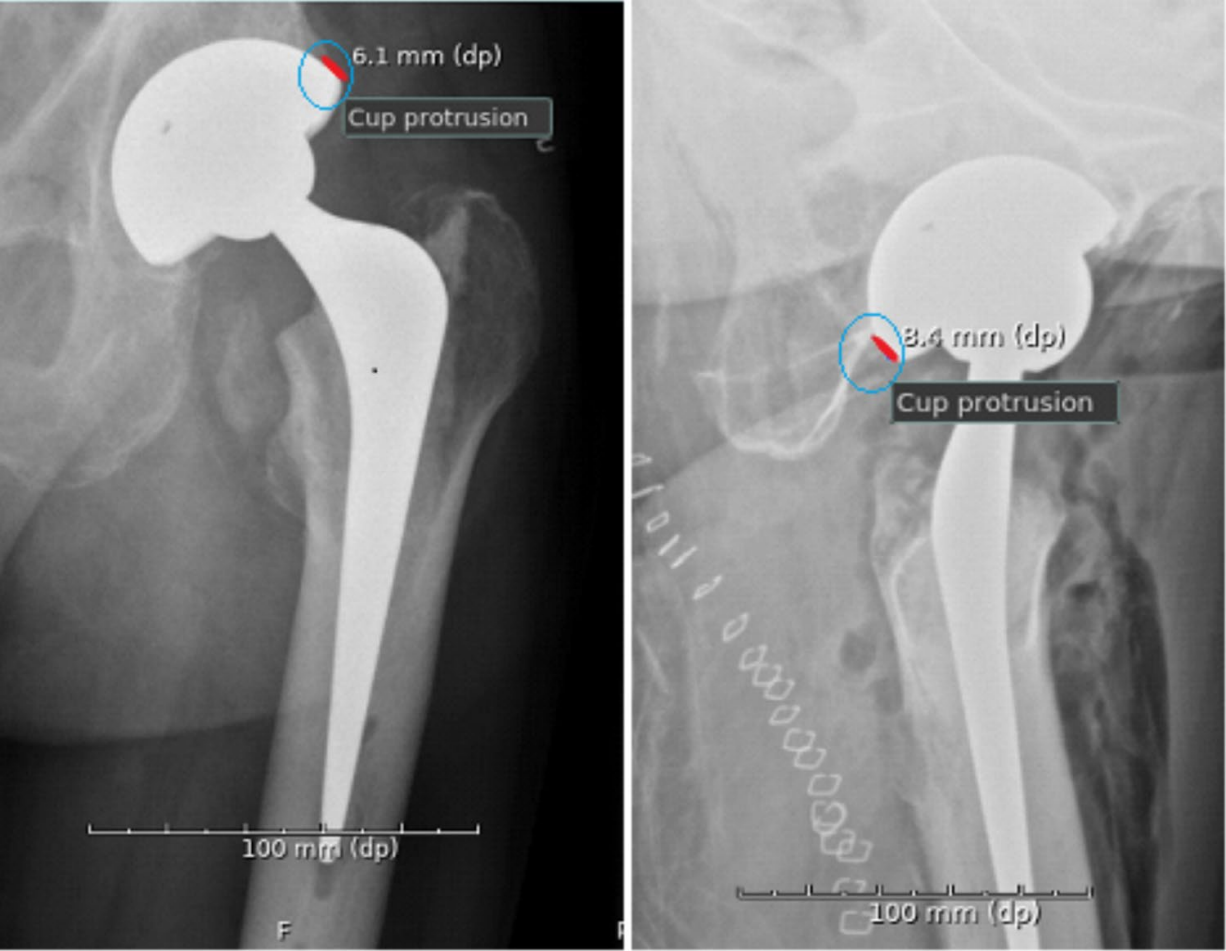
Cup protrusion measured on AP pelvic radiograph and a cross-table lateral radiographs

**Fig. 3:**
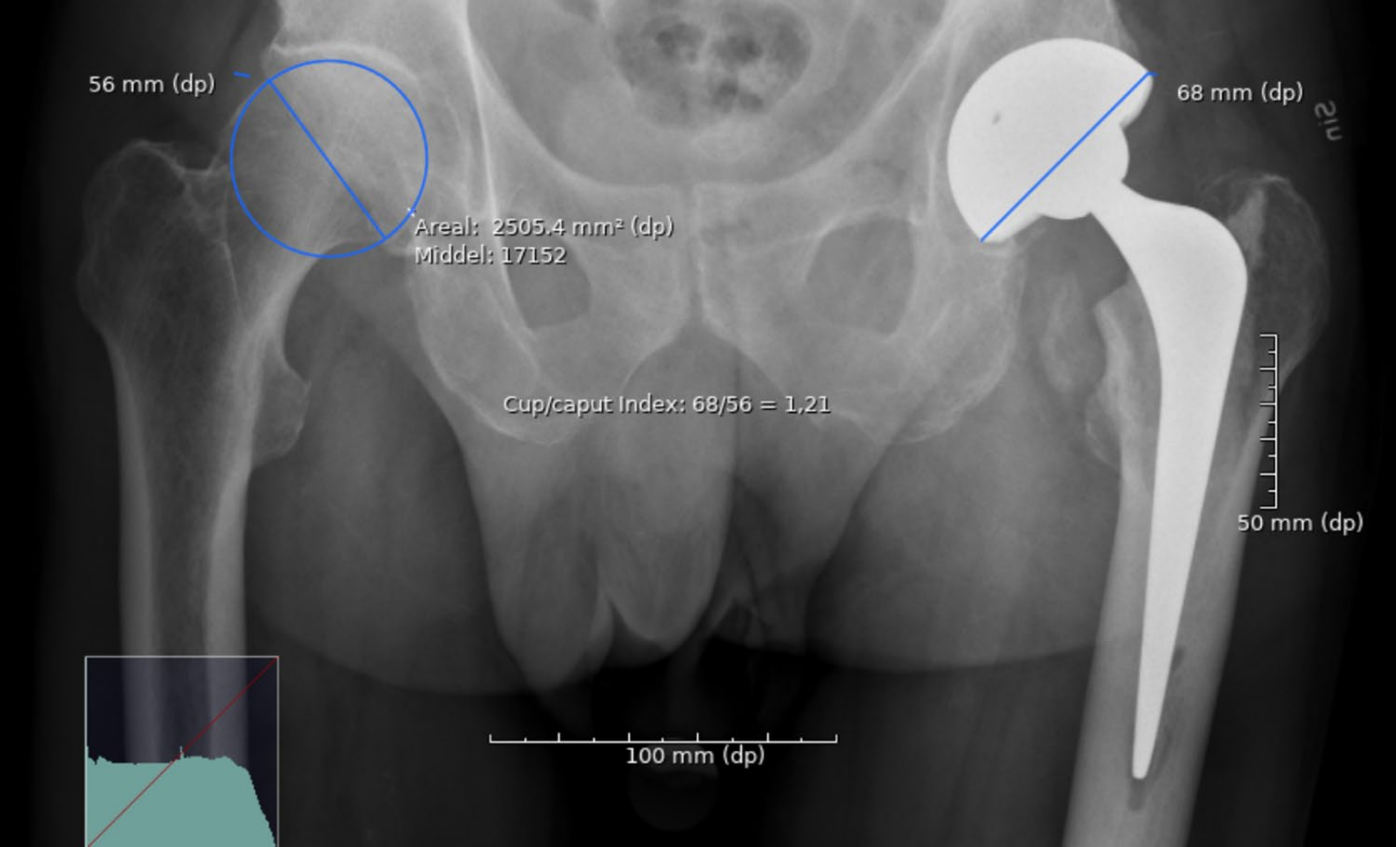
Cup/caput Index measured on AP pelvic radiograph

Digital postoperative AP pelvic and cross-table lateral X-rays were available for all patients. Digital X-rays were imported to the Mdesk^™^ system program and calibrated by caput size. A digital template provided by Mdesk^™^ was placed over the digital images and the following measurements were generated by the Mdesk^™^: Anteversion of the cup, inclination, hip center, leg length, cup protrusion, offset, and cup/caput index. Specific definitions for each measurement outcome are available in the Mdesk^™^ user manual. Mdesk^™^ version 4.0 was used for analysis (Mdesk^™^, RSA Biomedical Inc, Umea, Sweden) Visualization examples of the Mdesk^™^ template and selected measurement outcomes are present in Figs. 1, 2 and 3.

### Study Procedure

Raters were blinded to each other’s assessments and no preliminary Mdesk training was conducted to simulate real-world application scenarios.

Two independent senior orthopedic surgeons rated the x-rays (Raters A and B). Both raters were previously naive to the digital image analysis program Mdesk and no training was performed before the study started.

The X-rays of all patients were assessed twice with a wash-out period of at least two months by rater A (Rater A1 and A2). Rater B assessed the X-rays once (Rater B1).

### Statistical Analysis

Due to the study design no sample size calculation was performed. We consider a total of two raters and 102 cases to fulfill the study aim.

Continuous data were expressed as means and 95% confidence intervals (95%CI). The intra- and inter-tester reliability was reported by a two-way mixed model of interclass correlation coefficients (ICC) with 95% confidence intervals (CI) and absolute agreement. ICC values were interpreted as 0.0–0.3 low, 0.3–0.7 moderate, and 0.7–1.0 high. The standard error (SE) of the measurements was calculated using the ICC as SEmeas = SD × √1 – ICC. The ICC describe the extent to which two outcomes are likely to be similar.

For intra-test reliability, the smallest detectable change (SDC) was reported to represent the minimum change that needs to be observed to be confident that the observed change is real and not potential. The SDC was calculated as the standard error of the measurements × 1.96 × √2.

For inter-tester reliability, the 95% limits of agreement (LoA), estimated by mean difference ± 1.96 × standard deviation of the differences, was reported to provide an interval within which 95% of differences between measurements by the two raters are expected to lie (Rater A1, B1&A2, B1).

The statistical analysis used StataMP version 16 (StataCorp LLC, Texas).

## Results

A total of 102 patients were included in the study. The sample comprised 50 females and 52 males, aged 26–73 years (mean 58.6).

### Basic Characteristics of Implant Positioning

The mean cup inclination was 42.8° (95%CI 42.06–43.50), and the cup anteversion was 11.8° (95%CI 11.03–12.64). The mean Δ medial offset and Δ lateral offset were −4.5 mm (95%CI −5.12 to -3.94) and −1.2 mm (95%CI −1.98 to −0.51), respectively. The mean cup protrusion was 1.8 mm (95%CI: 1.48–2.13)) on AP pelvic radiographs and 0.9 mm (95%CI 0.42–1.23), and the mean cup/caput index was 1.2 (95%CI 1.16–1.18) (Table 1).

**Table 1:** Mean values (Tester A1(*N* = 102), A2 (*N* = 102) and B1 (*N* = 102))

Variable	Observations	Mean	SEmeas	95%CI
Cup inclination	306	42.8°	0.4	42.06-43.50
Cup anteversion	306	11.8°	0.4	11.03-12.64
Δ leg length	306	1.7mm	0.4	0.90-2.42
Δ medial offset	306	−4.5mm	0.3	−1.18
Δ lateral offset	306	−1.2mm	0.4	−1.47
Δ hip centre	306	4.2mm	0.3	3.66-4.82
Cup protrusion (AP)	306	1.8mm	0.2	1.48-2.13
Cup protrusion (lateral)	306	0.9mm	0.2	0.42-1.23
Cup/caput Index	306	1.2	0	1.16-1.18

### Intra-tester Reliability

The intra-tester reliability ranges from an ICC value of 0.99 (95%CI 0.991–0.996) when measuring cup inclination to 0.88 (95%CI 0.823–0.917) when measuring medial offset. The results indicate high intra-test reliability for all measurements, with the smallest detectable changes ranging from 0.1 to 1.1 (Table 2).

**Table 2:** Intra-tester reliability

	Measurement	ICC (95%CI)	SDC
Cup inclination	Intra-rater (A1 vs. A2)	0.994 (0.991–0.996)	1.1
Cup anteversion	Intra-rater (A1 vs. A2)	0.951 (0.929–0.967)	1.0
Δ leg length	Intra-rater (A1 vs. A2)	0.977 (0.967–0.985)	1.1
Δ medial offset	Intra-rater (A1 vs. A2)	0.88 (0.823–0.917)	0.8
Δ lateral offset	Intra-rater (A1 vs. A2)	0.953 (0.932–0.968)	1.0
Δ hip center	Intra-rater (A1 vs. A2)	0.969 (0.955–0.979)	0.8
Cup protrusion (AP)	Intra-rater (A1 vs. A2)	0.925 (0.891–0.949)	0.5
Cup protrusion (lateral)	Intra-rater (A1 vs. A2)	0.955 (0.933–0.969)	0.6
Cup/caput Index	Intra-rater (A1 vs. A2)	0.942 (0.915–0.96)	0.1

### Inter-tester Reliability

The inter-test reliability ranges from an ICC value of 0.98 (95%CI: 0.958–0.985) (A2-B1) for cup inclination to 0.60 (95%CI 0.458–0.712) for cup protrusion (A2-B1). The results indicate high inter-test reliability for the measurements inclination, anteversion, leg length, medial offset, hip center, protrusion (AP), and cup/caput index. Moderate inter-tester reliability was observed for the measurements of lateral offset (0.70) and cup protrusion (lateral) (0.60). The 95%LoA ranging from −7.2 to 12.1. (Table 3).

**Table 3:** Inter-tester reliability

	Measurement	ICC (95%CI)	95%LOG
Cup inclination	Inter-rater (A1 vs. B1)	0.971 (0.953–0.982)	−3.1;2.1
	IInter-rater (A2 vs. B1)	0.976 (0.958–0.985)	−3.4;2.4
Cup anteversion	Inter-rater (A1 vs. B1)	0.917 (0.879–0.943)	−6.2;5.9
	IInter-rater (A2 vs. B1)	00.909 (0.868–0.937)	−6.2;5.4
Δ leg length	Inter-rater (A1 vs. B1)	0.861 (0.801–0.904)	−6.2;7.0
	IInter-rater (A2 vs. B1)0	0.873 (0.818–0.912)-	−6.6;7.3
Δ medial offset	Inter-rater (A1 vs. B1)	0.916 (0.878–0.942)	−6.1;6.6
	IInter-rater (A2 vs. B1)	0.817 (0.741-0.873)-	−4.3;4.1
Δ lateral offset	Inter-rater (A1 vs. B1)	0.665 (0.405-0.802)	−7.2;10.9
	IInter-rater (A2 vs. B1)0	0.708 (0.559-0.806)	−6.5;12.1
Δ hip centre	Inter-rater (A1 vs. B1)	0.829 (0.757-0.881)	−5.7;5.6
	nIter-rater (A2 vs. B1)	0.836 (0.767-0.886)	−6.0-5.6
Cup protrusion (AP)	Inter-rater (A1 vs. B1)	0.732 (0.622-0.812)	−3.7;4.6
	IInter-rater (A2 vs. B1)	0.713 (0.601-0.798)	−3.5;4.6
Cup protrusion (lateral)	Inter-rater (A1 vs. B1)	0.614 (0.477-0.722)	−6.5;6.3
	IInter-rater (A2 vs. B1)0	0.600 (0.458-0.712)-	−6.5;6.1
Cup/caput Index	Inter-rater (A1 vs. B1)	0.736 (0.632-0.814)	−0,2;0.02
	IInter-rater (A2 vs. B1)0	0.754 (0.656-0.827)-	−0,-0, 1;0.1

## Discussion

This study showed high intra- and inter-tester reliability in THA patients for most key radiographic measurements of the acetabular component using the digital image analysis program Mdesk. Considering acetabular positioning is crucial for achieving optimal implant longevity and function following THA, and such information is highly important in clinical practice when using the Mdesk digital image program for postoperative analysis outcomes of THA. To the authors’ knowledge, this study is the first to report the reliability of a postoperative analysis of acetabular measurements derived from the commonly used digital analyzing system Mdesk.

The results from the present study showed a mean cup inclination and cup anteversion angle of 42.8° and 11.8°, respectively; the results are comparable to those reported in the literature, ranging from 37.5° to 49.7° for cup inclination and 10.7° to 27.3° for cup anteversion [14, 17, 19].

Previously, several studies have investigated the reliability of selected acetabular measurements based on other digital analyzing systems [20–22]. The present study showed high inter- and intra-tester reliability scores comparable to a study by Danoff et al.[20] reporting ICC values above 0.96 when measuring cup inclination and cup anteversion on 1289 patients with primary THA based on the HAS Version 8.0.4.1 (Martell Hip Analysis Suite™, Chicago, IL). Moreover, Shin et al.[21] evaluated the reliability and accuracy of measuring cup anteversion based on the methods reported by Liaw[22] and Woo and Morrey [23] and compared results to the PolyWare software (Draftware Developers Inc., Vevay, Indiana). The results from all three methods showed high reliability and were comparable to the present study.

The femoral offset (FO) was measured as Δ medial offset and Δ lateral offset. The measurement was performed bilaterally to compare the femoral offset on the operated side to the non-operated hip. Intra-tester ICC values of 0.88 (0.823–0.917) and 0.953 (0.932–0.968) for Δ medial offset and Δ lateral offset indicated high intra-tester reliability. This is similar to the findings of Mahmood et al. [13] who compared the Sundsvall method of FO measurements (ICC 0.94 (95%CI 0.91–0.96)) with the standard method (ICC 0.94 (95%CI 0.90–0.96)).

The inter-tester ICC values of Δ lateral offset and cup protrusion from the present study were observed with moderate reliability. Compared to the inter-tester reliability of lateral offset reported by Mahmood et al.[13], findings for the present study were slightly lower. ((ICC 0.92 (95%CI: 0.89–0.94) for the Sundsvall method and ICC 0.88 (95%CI 0.84–0.91) for the standard method). This difference may be due to the difference in radiological technique for obtaining X-rays between studies. The existing literature included limited information regarding postoperative analysis derived for the Mdesk system.

In the clinical setting, the present findings indicate that post-operative X-rays can be used to evaluate Δ medial offset, but caution should be used when evaluating cup protrusion and Δ lateral offset. More research is needed.

This study had several limitations. First, the study lack comparison to CT scan. Recent studies have shown that CT scans are superior to radiographs in measuring acetabular position. The combination of CT scans and radiographic information is likely more accurate in measuring cup position [19]. However, in the authors’ opinion, the clinical implications of a slightly more accurate method of measuring inclination and version might not outweigh the more costly examination and added X-ray dose of the CT scan. Furthermore, it is likely that standard X-rays will still be the most commonly used tool for postoperative examination of cup positioning in the foreseeable future. Further studies of the Mdesk comparing radiograph vs. CT scan accuracy may be needed for further validation.

The focus of the present study was on the acetabular component; knowing the positioning of the femoral component also impacts complications and survival of the construct following THA.

Analyzing radiographs using only one software program might not be transferable to other software systems. However, studies comparing different software systems reveal high comparability [14].

In our study, two senior orthopedic surgeons familiar with total hip arthroplasty surgery analyzed the radiographs using a digital image analysis program. This fact might limit the external validity of the present study. More research is needed to establish if younger surgeons or other professions can achieve comparable results.

Given the limitations, this study shows that Mdesk might be a useful clinical tool with high reliability for clinical monitoring cup anteversion and inclination following total hip replacement.

Moreover, studies comparing Mdesk reliability to CT-based methods or alternative digital radiographic systems are needed.

## Conclusion

This study showed high intra- and inter-tester reliability for most key radiographic measurements of the acetabular component following unilateral THA using the digital image analysis program Mdesk. The results indicate that the digital analysis program Mdesk may be useful in clinical practice for postoperative acetabular quality monitoring following THA.

## Data Availability

The raw data are available with the corresponding author.
